# Breaking Immunological Tolerance in Systemic Lupus Erythematosus

**DOI:** 10.3389/fimmu.2014.00164

**Published:** 2014-04-09

**Authors:** Elmar Pieterse, Johan van der Vlag

**Affiliations:** ^1^Department of Nephrology, Radboud University Medical Center, Nijmegen, Netherlands

**Keywords:** systemic lupus erythematosus, apoptosis, neutrophil, NETosis, histone modifications

## Abstract

Systemic lupus erythematosus (SLE) is a fairly heterogeneous autoimmune disease of unknown etiology that mainly affects women in the childbearing age. SLE is a prototype type III hypersensitivity reaction in which immune complex depositions cause inflammation and tissue damage in multiple organs. Two distinct cell death pathways, apoptosis and NETosis, gained a great deal of interest among scientists, since both processes seem to be deregulated in SLE. There is growing evidence that histone modifications induced by these cell death pathways exert a central role in the induction of autoimmunity. In the current review, we discuss how abnormalities in apoptosis, NETosis, and histone modifications may lead to a break of immunological tolerance in SLE.

## Introduction

Autoimmune disorders are disturbances of the immune system that arise when the immune system responds to self. Immunological tolerance to self relies on the immune system to discriminate self from non-self. Systemic lupus erythematosus (SLE) is a systemic autoimmune disorder, in which primarily nuclear constituents, i.e., DNA, histones, and ribonucleoproteins, are targeted (1). In a substantial subset of SLE patients, autoantibodies also target proteins from the cytoplasm of neutrophils (2). Since these nuclear self-antigens are normally shielded from the extracellular space by the nuclear membrane and the cell membrane, the key question is how these nuclear self-antigens are released and become exposed to the immune system. Anno 2014 abnormalities in two pathways of cell death, apoptosis and NETosis, are recognized as central processes that provide nuclear autoantigens and drive the autoimmune response in SLE (3).

## Two Sources of Autoantigens in SLE

For over 20 years, apoptosis has been considered as the major source of autoantigens in SLE (4). Apoptosis is a highly organized and immunologically silent cell death pathway that plays an important role in tissue homeostasis. In processes characterized by a high-rate of tissue turnover, such as embryogenesis in human development, apoptosis is a crucial mechanism that allows tissues to remodel without triggering inflammation. Many cellular pathways and signals can activate proteolytic caspases to break down the cell in a strictly controlled and fine-tuned manner that distinguishes apoptosis from any other form of cell death. Apoptosis can be induced actively through ligation of cell surface receptors such as Fas or TNFR or passively through lack of essential survival signals. Apoptotic cells undergo a series of distinct morphological changes, including cytoskeletal disruption, cell shrinkage, DNA fragmentation, and plasma membrane blebbing (5). It has been shown that many of the nuclear autoantigens targeted in SLE are concentrated within apoptotic blebs (6, 7).

A specialized form of neutrophil cell death, termed NETosis, has been described a decade ago (8). NETosis has been linked to SLE as an additional source of autoantigens (9). During NETosis, neutrophils extrude fibrillary networks composed of DNA, citrullinated histones, and granule peptides such as neutrophil elastase, myeloperoxidase, and cathepsin G. These structures are termed neutrophil extracellular traps (NETs) and serve to entrap and dismantle not only extracellular bacteria, but also viruses, fungi, and parasites (10–12). In addition to pathogens, sterile inflammatory mediators such as monosodium urate (MSU) crystals, IL-8, IL-1β, platelet-activating factor (PAF), and TNF-α have been reported to induce NETosis (13). NETosis requires a very rapid disintegration of the nuclear envelope, translocation of granule peptides to the nucleus, PAD4-mediated citrullination of the chromatin, binding of granule peptides to citrullinated chromatin, and finally rupture of the plasma membrane (14). Where apoptosis is organized and planned, NETosis seems much faster and less well-coordinated.

## Increased Cell Death

MRL/lpr mice, the most commonly studied murine model for lupus-like disease, develop an autoimmune disease that reflects pathologies of human SLE, including lymph node enlargement, increased IgG levels, antinuclear autoantibody production, glomerulonephritis, proteinuria, and development of skin lesions (15). MRL/lpr mice express a defective form of the Fas receptor that under physiological conditions stimulates cells to undergo apoptosis. This initially led to the belief that SLE patients have a similar defect in Fas-mediated apoptosis that underlies the failure of self-tolerance. However, it has become clear that SLE is quite the opposite from being a disease with impaired apoptosis. Substantially, evidence correlates increased lymphocyte, neutrophil, macrophage, and monocyte apoptosis directly to SLE disease activity (16–19). It has been demonstrated that SLE serum has a strong apoptosis-inducing capacity in macrophages, monocytes, and lymphocytes from healthy donors (20). In addition, it has been reported that autoreactive T cells show an increased expression of the apoptotic ligands TRAIL, TWEAK, and FasL that directly mediate the apoptosis of monocytes (21).

Since various bactericidal NET proteins were found to be present at much higher levels in blood from patients with SLE compared to healthy donor blood, enhanced NETosis is also implicated in the genesis and/or amplification of the autoimmune response in SLE (22–24). The pro-inflammatory cytokines IL-17A, TNF-α, IL-1β, and IL-8 have all been reported to induce NETosis, suggesting that NETs are extensively formed in an inflammatory environment such as in SLE (25–27). Elevated levels of interferon-alpha (IFN-α), a cytokine that under physiological conditions is important for antiviral responses and immune activation, have been correlated with disease activity in SLE (28). Recent evidence indicates that NETosis is enhanced in SLE due to priming of neutrophils by IFN-α (29). Certain autoantibodies that are highly present in the majority of SLE patients, such as anti-LL-37, anti-HNP, and anti-RNP, can also stimulate neutrophils to produce NETs in a FcγRIIa-dependent manner (30–32). Furthermore, a distinct subset of neutrophils in SLE patients, so called low-density granulocytes (LDGs), have been described with an increased NET-releasing capacity due to their ability to synthesize IFN-α in an autocrine manner (33).

Taken together, it is presumed that both apoptosis and NETosis occur excessively in patients with SLE, which results in an increased load of nuclear autoantigens. However, can this excessive release of nuclear autoantigens explain the break of immunological tolerance to nuclear antigens in SLE? Apoptosis and NETosis are physiological forms of cell death. In our daily battle against pathogens, we release NETs and between 50 and 70 billion cells die daily due to apoptosis. Humans evolved redundant mechanisms to clear apoptotic material and NETs. This clearance is usually accompanied by secretion of anti-inflammatory cytokines (34). Multiple groups tried to immunize mice with apoptotic cells/blebs or NETs, but this never lead to considerable immune activation (35, 36). Therefore, it can be concluded that increased apoptosis or NETosis on its own is not sufficient to break immunological tolerance to nuclear autoantigens in SLE, and that additional factors are required to turn apoptotic material or NETs into dangerous triggers of autoimmunity.

## Clearance Deficiency in SLE

In 1980, it was for the first time described that macrophages from SLE patients show an impaired phagocytic activity for yeast (37). Later, it was described that the phagocytosis of autologous apoptotic material by monocyte-derived macrophages is also disturbed in about 50% of the SLE patients (38). This finding was confirmed by other groups (18, 39). Interestingly, macrophages differentiated from CD34 positive stem cells of SLE patients show a different morphology than those generated from healthy donors; they are relatively small and poorly ingest apoptotic material (40). Furthermore, the amount of tingible body macrophages (TBMs) found in germinal centers appears to be strongly reduced in SLE patients (41). Apoptotic material in germinal centers is normally internalized by TBMs. In SLE patients with a reduced number of TBMs, apoptotic material was observed to be directly associated with the surface of follicular dendritic cells (FDCs), which may provide survival signals for autoreactive B cells. Monocytes and granulocytes from SLE patients display a reduced phagocytic activity as well (42), which may be explained by the relative low expression of the phagocytic receptor CD44 on both cell types (43).

In addition to well-functioning healthy phagocytes, serum proteins have an important impact on the clearance of apoptotic cells as well. Adequate removal requires a clear recognition of apoptotic cells, which is, in addition to the exposure of phosphatidylserine on the outer leaflet of its plasma membrane, strongly dependent on opsonizing proteins such as immunoglobulin M (IgM), mannose-binding lectin (MBL), serum amyloid P (SAP), C-reactive protein (CRP), and C1q. Numerous studies ascribe a role for these opsonins in the defective clearance of apoptotic material in SLE. It has been shown that decreased IgM levels and increased MBL levels correlate with an increased disease activity in SLE (44, 45), that administration of SAP and CRP significantly delays disease onset and alleviates disease symptoms (46, 47) and that C1q-deficient mice rapidly develop autoantibodies against nuclear autoantigens (48). Polymorphisms at the loci of the genes encoding these opsonins as well as the formation of autoantibodies against these opsonins are considered to be the underlying cause for their absence or defective functioning in SLE (49–52). Lastly, autoantibodies against pentraxin-related protein PTX3, a cytokine-induced protein that is homologous to CRPs and SAPs, appear to be frequently present in SLE patients as well (53). In contrast to anti-CRP or anti-SAP autoantibodies, anti-PTX3 autoantibodies are not associated with disease activity but with the absence of glomerulonephritis and antiphospholipid antibodies. The authors of this article suggest that PTX3 inhibits the clearance of apoptotic material, which is counteracted by the autoantibodies directed against them.

There is also evidence for an impaired clearance of NETs. Deoxyribonuclease I (DNase I) plays a crucial role in the degradation of NETs, which is not surprising since the backbone of NETs is composed of nuclear DNA. The relevance of proper DNase I activity is reflected by the fact that DNase I-deficient mice develop a syndrome that closely resembles to SLE (54). A considerable number of SLE patients display a reduced DNase I activity (55). These patients develop relative high titers of anti-dsDNA autoantibodies and suffer from more severe symptoms. Low DNase I activity in SLE may have a genetic cause (56) but can also be the result of inhibitory molecules or anti-DNase I autoantibodies. In a Taiwanese cohort, 62% of the SLE patients appeared to be positive for anti-DNase I autoantibodies compared to only 8% of normal controls (57). Comparing sera from healthy donors and patients with SLE in their capability of degrading NETs *in vitro* revealed that 98.1% of the healthy donor sera degraded NETs normally, whereas a significant percentage of the SLE sera did not (36.1%) (58). Interestingly, those patients who could not degrade NETs developed lupus nephritis significantly more frequently than those who could degrade NETs. Depleting autoantibodies from SLE sera considerably enhanced NET degradation, suggesting that NET-bound autoantibodies inhibit NET degradation, most likely by preventing the access for DNase I to the NET. In addition, it was shown that NET-bound C1q also inhibits NET degradation (59) via the same mechanism. However, C1q seems to be a double-edged sword in the removal of NETs: both recombinant C1q and endogenous C1q derived from human serum were found to opsonize NETs for their immunologically silent clearance by macrophages (60). Lastly, it has been shown that the antimicrobial peptides LL-37 and HMGB1 prevent NET degradation (61). This latter observation is interesting, since it has been described that these antimicrobial peptides are highly present in NETs from SLE patients but not in those from healthy donors (32).

The contribution of intrinsic phagocyte defects and absent/deficient serum factors to the impaired clearance of apoptotic material and/or NETs in SLE seems clear. However, it is believed that there are numerous additional factors and pathways that could play a role in the complex pathogenesis of SLE. Although many different pathways may be deregulated, that all lead to a comparable SLE phenotype, it is assumed that accumulation of apoptotic material and NETs in tissues is the common denominator between all patients with SLE (42). Nevertheless, the question remains how such an accumulation of apoptotic material and NETs can break immunological tolerance. After all, it involves an accumulation of endogenous material that is not supposed to elicit an autoimmune response. An important hypothesis that gains growing support states that biochemical reactions, for example cleavage by caspases or protein modifications by protein modifying enzymes, lead to enrichment of protein modifications in not efficiently cleared NETs or apoptotic cells (62). Certain (combinations of) protein modifications may give rise to proteins with neoantigens that behave as danger signals as well and thereby are no longer perceived as endogenous and therefore have the ability to initiate an autoimmune response. Neoantigens/danger signals in NETs are directly exposed to the immune system, but apoptotic blebs require to undergo secondary necrosis, a late apoptotic stage characterized by loss of membrane integrity and leakage of cellular constituents, for exposure of these neoantigens/danger signals to the immune system (63). Neoantigens/danger signals may be ingested, digested, and presented in an immunogenic way by antigen-presenting cells in MHC class II to autoreactive CD4+ T cells, who subsequently instruct autoreactive B cells to produce autoantibodies against them. Alternatively, extracellular autoantigens may be presented in MHC class I, via the mechanism of cross-presentation, to autoreactive CD8+ T cells (64). The concept of epitope spreading may ultimately lead to the wide arsenal of autoantibodies that are characteristic for SLE. An important group of proteins in which post-translational modifications (PTMs) seem to play an important role in (the induction of) autoimmune responses in SLE are histones (65).

## Histone Modifications Related to SLE

**Figure 1:**
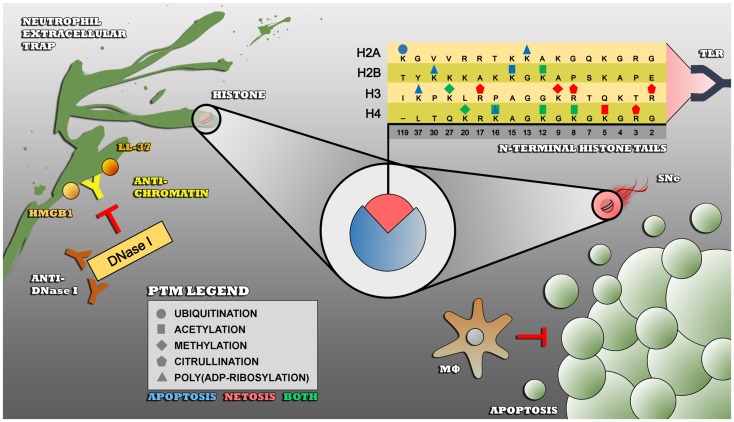
**Impaired clearance of apoptotic cells and/or NETs leads to an enduring exposure of modified histones to the immune system – insufficiently cleared apoptotic cells by macrophages undergo secondary necrosis (SNe), thereby externalizing modified autoantigens such as histones that become recognized as foreign and dangerous by receptors of the innate immune system such as toll-like receptors (TLR)**. Modified histones are also highly present in NETs, that are also not properly cleared in SLE due to polymorphisms in the DNase I gene (not shown), inhibitory anti-DNase I autoantibodies, or NET-bound proteins such as HMGB1, LL-37, C1q (not shown), and anti-chromatin autoantibodies that prevent the accessibility for DNase I to the NET. The PTMs that are shown are associated with apoptosis (blue), NETosis, or both (green) and are linked to the autoimmune response in SLE.

Histones are a group of chromatin proteins that are abundantly present in apoptotic blebs as well as in NETs. Anti-histone autoantibodies are frequently found in SLE and are disease-specific (66). Under physiological conditions, histones play a critical role in the packaging of nuclear DNA. Eukaryotic cells possess five major families of histones: H1/H5, H2A, H2B, H3, and H4. Histones H2A, H2B, H3, and H4 are known as the core histones: two copies of each of the four core histones assemble and are wrapped with ~146 bp of DNA to form the fundamental unit of chromatin known as the nucleosome. Histones were originally thought to solely function as a static scaffold for DNA packaging. Nowadays, it is evident that histones are highly dynamic proteins, undergoing multiple types of PTMs that regulate vital processes within the cell such as transcription, replication, recombination, and DNA repair. PTMs on histones mainly occur at the N-terminal tails and include (but are not limited to) acetylation, methylation, ubiquitination, poly(ADP-ribosyl)ation, and citrullination. These PTMs are described below, with a focus on modifications associated with cell death, which have been specifically related to autoimmune situations such as SLE (Figure 1).

### Acetylation

Acetylation and deacetylation of lysine residues at the N-terminal tails of histones play an important role in the regulation of transcription. Acetylation removes positive charges, thereby reducing the affinity between histones and DNA and maintaining an open and accessible conformation of DNA that is available for the binding of factors of the transcriptional machinery. Acetylation and deacetylation reactions are catalyzed by enzymes with respectively histone acetyltransferase (HAT) or histone deacetylase (HDAC) activity. Various studies have investigated the significance of histone acetylation in SLE. Hypoacetylation of histones H3 and H4 has been found in CD4+ T cells from SLE patients and splenocytes from MRL/lpr mice (67, 68). Treatment of these MRL/lpr mice with HDAC inhibitor trichostatin A reset the hypoacetylation of histones H3 and H4 and lead to an improved disease phenotype. In addition, it has been shown that mice deficient in HAT p300 develop an autoimmune disease similar to SLE in its pathological manifestations (69). Altered histone acetylation in unstimulated disease-relevant cells has been primarily linked to an altered gene expression. In contrast to unstimulated cells, apoptotic blebs or NETs from SLE patients contain hyperacetylated histones when compared to healthy donors (submitted data of our group). Hyperacetylation of histones appears to occur early during cell death. In the past, our group showed that hyperacetylated nucleosomes and acetylated histone peptides display an enhanced capability in activating the immune system (70). Hyperacetylated nucleosomes were able to mature bone marrow-derived DCs *in vitro*, which produced increased levels of IL-6 and TNF-α compared with DCs cultured in the presence of normally acetylated nucleosomes. In addition, DCs cultured in the presence of hyperacetylated nucleosomes were able to activate syngeneic T cells. Furthermore, subcutaneous administration of a specific tri-acetylated H4 peptide to pre-diseased MRL/lpr mice significantly enhanced mortality, proteinuria, skin lesions, and glomerular IgG depositions. In addition to a direct immunogenic affect, it is speculated that hyperacetylated histones in apoptotic blebs or NETs enhance the recruitment and binding of “dangerous” antimicrobial peptides such as LL-37, HMGB1, and HNPs. It has been shown that these antimicrobial peptides display a high immunogenic potential in NETs (61). Our group showed that autoantibodies in SLE patients frequently target acetylated epitopes in the N-terminal tails of histones H2A, H2B, and H4. We characterized two monoclonal autoantibodies derived from a MRL/lpr mouse, KM-2 and LG11-2, that recognize tri-acetylated H4 at lysines 8, 12, and 16 and acetylated H2B (K12), respectively (70, 71). Both autoantibodies showed an increased reactivity with histones isolated from apoptotic cells and also from NETs (submitted data of our group), suggesting that these modifications are associated with apoptosis and NETosis. Another study also showed that IgG autoantibodies from histone-reactive SLE patients show high reactivity for acetylated H2B, whereas an increase in H4 acetylated at lysine 5 in NETs was demonstrated as well (36).

### Methylation

Histone methylation is the process by which methyl groups are transferred by histone methyltransferases (HMTs) to lysine or arginine residues of histones. Similar to acetylation, histone methylation regulates transcription and silencing of genes, depending on the target sites. Di-methylation of H3 at lysine 9 and mono-, di-, and tri-methylation of H4 at lysine 20 increases upon NETosis (36). Methylation of histone H4 at lysine 20 has also been associated with apoptosis (72). Our group recently demonstrated that autoantibodies present in the plasma from SLE mice and patients preferentially recognize tri-methylated H3 at lysine 27 (73). This latter reactivity was specific for SLE as there was hardly any reactivity in plasma samples from patients with rheumatoid arthritis (RA) or systemic sclerosis and healthy controls. Tri-methylation of H3 at lysine 27 also increases upon NETosis, as demonstrated by Liu et al. in ATRA-differentiated HL-60 cells (36). In our hands, this epitope is also highly present in NETs formed by primary neutrophils (submitted data of our group). Methylation of histones seems to be associated with an increased immunogenic potential of chromatin, similar to the aforementioned acetylation of histones, but additional research is required to unravel the exact mechanisms.

### Ubiquitination

The process of ubiquitination involves the conjugation of ubiquitin to other cellular proteins, thereby regulating a broad range of eukaryotic cell functions such as apoptosis, antigen processing, DNA transcription and repair, cell division, and immune responses. Ubiquitination may signal proteins for their degradation via the proteasome, alter their cellular location, affect their activity, and promote or prevent protein–protein interactions. In human, 10% of all H2A proteins is monoubiquitinated at lysine 119 (UH2A) (74). Autoantibodies against UH2A are frequently found in SLE and appear to be disease-specific (75). Between 60 and 70% of SLE patients are positive for anti-UH2A autoantibodies, compared to 10% of patients with systemic sclerosis. In RA, juvenile chronic arthritis, or Sjögren’s syndrome, these autoantibodies are virtually absent. Deposits of UH2A have been identified in more than 50% of the renal biopsies from SLE patients with glomerulonephritis (76). Disappearance of UH2A (deubiquitination) is linked to late apoptotic processes and is likely to be disturbed in SLE (77, 78). Polymorphisms in the TNFAIP3 gene, the gene that encodes for the deubiquitinating enzyme A20, are highly associated with SLE (79). These polymorphisms lead to a reduced expression of A20 and result in increased ubiquitination, as demonstrated by Jury et al. in T cells from SLE patients (80). Due to the inhibitory effect of A20 on the NFκB signaling pathway, TNFAIP3 polymorphisms also cause hyperactive NFκB signaling. The ribonucleoprotein SSA/Ro is, in addition to UH2A, also a ubiquitinated protein that is frequently target of SLE autoantibodies (81). Hyperubiquitinated histones released from late apoptotic cells or NETs are likely to display an increased antigenicity and immunogenicity, but the underlying mechanisms are not fully elucidated yet.

### Poly(ADP-ribosyl)ation

Poly(ADP-ribosyl)ation of proteins involves the addition of poly(ADP-ribose) moieties (PARs), mediated by poly(ADP-ribose) polymerases (PARPs). These reactions are involved in cell signaling and the control of many cell processes, including DNA repair, telomere maintenance, and apoptosis. Autoantibodies that bind to PARs or to the two zinc finger motifs of PARPs are frequently found in patients with autoimmune rheumatic and bowel diseases, and SLE (82–84). Anti-PARP autoantibodies do not significantly affect the enzyme activity of PAPRs, but prevent the cleavage of PARPs by caspase-3 (85). This cleavage is important in the proper execution of apoptosis. Inefficient cleavage of PARPs has shown to prolong cell survival *ex vivo* and may therefore cause failure to eliminate autoreactive lymphocytes and sustain autoimmune stimulation. Anti-PARP autoantibodies can penetrate cells in relative late stages of apoptosis, thereby neutralizing PARP activity. As a result of energy depletion, prolongation of cell survival may ultimately result in necrosis, thereby releasing poly(ADP-ribosyl)ated proteins that may possess high antigenic and immunogenic potential. It has been shown that oligo(ADP-ribosyl)ated histones are involved in the production of anti-PARP autoantibodies in SLE patients (86). Lysine 13 of H2A, lysine 30 of H2B, lysine 37 of H3, and lysine 16 of H4 have all been identified as ADP-ribose acceptor sites (87).

### Citrullination

Citrullination (also known as deamination) involves the conversion of arginine residues into citrulline, a process catalyzed by enzymes known as peptidylarginine deiminases (PADs). While most of the previously discussed PTMs are reversible and associated with reversible events involved in signal transduction, citrullination is not reversible. Citrullination has an important role in the normal function of the immune system, skin keratinization, the insulation of neurons, and the plasticity of the central nervous system including its essential role in gene regulation. In RA, autoantibodies to citrullinated proteins (anti-CCP) is considered a key pathogenic event. The presence of anti-CCP autoantibodies is a powerful biomarker that allows the diagnosis of RA to be made at a very early stage (88). Many groups investigated the role of NETs in the production of anti-CCP autoantibodies in RA- and PAD4-mediated citrullination of histones, which appear as the essential initiator for NETosis via decondensation of the chromatin (89). Khandpur et al. correlated accelerated NETosis in RA with anti-CCP levels (25), but this correlation has raised the following question: if NETosis, initiated by citrullination of histones, plays a pathogenic role in both RA and SLE, then why is the presence of anti-CCP autoantibodies highly sensitive and specific for RA only and not for SLE? Hence, anti-CCP autoantibodies are present in only 10–30% of SLE patients compared to 80–90% of RA patients (90, 91). Interestingly, SLE patients with arthritis are significantly more positive for anti-CCP than those without arthritis, suggesting that these autoantibodies have a predictive value for the development of arthritis in SLE (92). A groundbreaking publication by Romero et al. questions the contribution of NETs to anti-CCP production (93). This group showed that proteins highly present in RA synovial fluid cells become hypercitrullinated due to membranolysis of these cells by perforins and the membrane attack complex (MAC) of the complement system. This membranolysis results in a massive calcium influx that activates PADs to citrullinate various substrates, such as vimentin, fibronectin, and α-enolase. Although it seems that citrullinated histones, present in NETs and to a lesser extent also in apoptotic blebs, do not exert an important role in the induction of anti-CCP autoantibody production, they may still play a pathogenic role. To our knowledge, studies about the immunogenic effect of citrullinated histones in NETs or apoptotic cells have not yet been conducted. The antimicrobial peptide LL-37 is highly present in NETs from SLE patients and has recently been found to be also a substrate of PADs (94). Citrullinated LL-37 showed to be more chemotactic to PBMCs and more pro-inflammatory compared to unmodified LL-37. Comparable results may also hold for citrullinated histones, although additional research is required.

## Concluding Remarks

**Figure 2:**
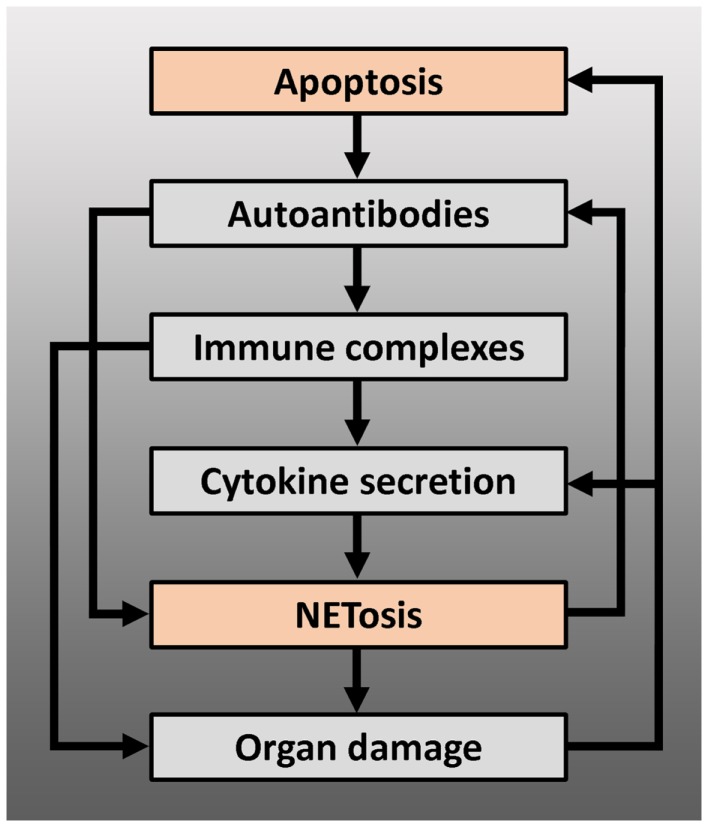
**Positive feedback loops arising from the interaction between apoptosis and NETosis, leading to chronification and/or exacerbation of the disease – modified autoantigens, derived from apoptotic cells, may be presented by antigen-presenting cells to autoreactive T cells, which can lead to production of autoantibodies by B cells, including anti-dsDNA or anti-RNP antibodies**. These autoantibodies can induce NETosis or form immune complexes with their antigen. Immune complexes deposit on basal membranes, and incite a local inflammation (organ damage), or stimulate plasmacytoid dendritic cells to produce IFN-α and other pro-inflammatory cytokines. Pro-inflammatory cytokines such as IL-1β, TNF-α, or IFN-α induce NETosis or prime neutrophils for NETosis: NETs may serve as B cell autoantigens and lead to further autoantibody production or directly cause organ damage. Proteins from neutrophil granules, present in NETs, have shown to be highly toxic to glomerular structures and endothelium. Endothelial or glomerular damage causes further production of pro-inflammatory cytokines and leads to a new load of apoptotic cells.

The exact etiopathogenesis of SLE is far from understood. Many different environmental factors are believed to act together to induce SLE in those who are genetically predisposed. There is a growing body of evidence that shows that disturbances in two cell death pathways, apoptosis and NETosis, are causative for initiating the disease and amplifying existing disease. However, the relative contribution of apoptosis and NETosis to the genesis of SLE is unclear and is likely to differ from patient to patient. Regardless of this, it is assumed that disturbances in both cell death pathways interact with each other and create multiple positive feedback loops that lead to chronification or exacerbation of the disease (Figure 2). Despite the heterogeneity in the underlying (molecular) defects and pathways that cause SLE, it appears that accumulation of apoptotic material and NETs in tissues is the common denominator between all patients with SLE. Enrichment of protein modifications, and in particular specific histone modifications, in not efficiently cleared apoptotic cells or NETs may generate neoantigens/danger signals with an increased antigenic and immunogenic potential. In addition to PTMs, another process to be considered in the generation of neoantigens/danger signals is proteolytic cleavage of histones and other chromatin-associated proteins by for example caspases, neutrophil elastase, and/or cathepsins. It is conceivable that chromatin-derived PTMs and/or cleavage products, related to apoptosis and/or NETosis, specifically ligate to receptors on antigen-presenting cells, thereby activating these cells and resulting in their immunogenic presentation. Improving or intensifying the clearance of apoptotic cells and/or NETs may prevent the formation of immunogenic nuclear autoantigens. In addition, neutralizing and tolerizing strategies using specific chromatin-derived PTMs and/or cleavage products related to apoptosis and/or NETosis may represent future therapies for SLE.

## Conflict of Interest Statement

The authors declare that the research was conducted in the absence of any commercial or financial relationships that could be construed as a potential conflict of interest.
